# Laboratory Monitoring of UFH in Different Settings (DEXHEP Study): Association between Anti-Xa Levels, Platelet Factor 4 (PF4) Plasma Levels and Dextran Sulfate

**DOI:** 10.1055/a-2749-6046

**Published:** 2025-12-02

**Authors:** Philippe Savard, Emmanuel Curis, Isabelle Gouin-Thibault, Marie Toussaint-Hacquard, Céline Delassasseigne, Anne Bauters, Claire Flaujac, Valérie Eschwège, Christine Mouton, Thomas Lecompte, Emmanuel de Maistre, Dominique Lasne, Virginie Siguret

**Affiliations:** 1 Centre Hospitalier Universitaire de Dijon (CHU Dijon), DijonFrance; 2 UR 7537 BioSTM, Université Paris Cité & Hôpital Lariboisière, ParisFrance; 3 Centre Hospitalier Universitaire de Rennes (CHU Rennes), RennesFrance; 4 Centre Hospitalier Régional Universitaire de Nancy (CHRU Nancy), NancyFrance; 5 Centre Hospitalier Universitaire de Bordeaux (CHU Bordeaux), BordeauxFrance; 6 Centre Hospitalier Régional Universitaire de Lille (CHU Lille), LilleFrance; 7 Centre Hospitalier de Versailles (CH Versailles), VersaillesFrance; 8 Faculté de Médecine, Université de Lorraine, NancyFrance; 9 UMR-S-1176, Hôpital Necker—AP-HP, ParisFrance; 10 UMR-S-1144, Hôpital Lariboisière—AP-HP, ParisFrance

**Keywords:** anti-Xa assays, unfractionated heparin, dextran sulfate, PF4

## Abstract

**Background:**

Chromogenic anti-Xa assay is currently used in the management of patients on unfractionated heparin (UFH). It has been shown that inter-assay variability in anti-Xa levels can be explained in part by the presence or absence of dextran sulfate (DXS) in the reagents. DXS has the ability to dissociate UFH from neutralizing proteins, including platelet factor 4 (PF4).

**Aims:**

Investigate whether PF4 plasma levels along with the presence/absence of DXS in anti-Xa reagents are associated with variations in UFH anti-Xa levels in different clinical situations.

**Methods:**

In the prospective multicenter study DEXHEP-NCT04700670, critically ill patients on UFH therapy (four groups) were recruited. Blood was collected into citrate and CTAD tubes. Chromogenic anti-Xa levels were assessed using seven reagent/analyzer combinations including two without DXS. Plasma PF4 was measured by ELISA (Zymutest-PF4-Hyphen-Biomed).

**Results:**

A total of 144 patients were analyzed: average PF4 levels in citrate plasma samples were consistently higher than in CTAD ones (206 vs. 46 ng/mL, *p* < 10 ^−4^ ), regardless of the patient group. Using a linear mixed-effect model, we found a significant effect of both DXS and PF4 on anti-Xa level, with a significant interaction term ( *p* < 10 ^−4^ ). Considering the 0.3 to 0.7 IU/mL therapeutic range, agreement between anti-Xa values (Liquid-anti-Xa/DXS-free vs. Biophen-LRT/DXS) was observed in roughly two-thirds of the patients.

**Conclusion:**

PF4 levels slightly affects anti-Xa levels, the use of CTAD tubes minimizing the effect. However, PF4 levels do not fully explain the differences of anti-Xa levels observed in the presence or absence of DXS, which has a greater effect. Anti-Xa assays require better standardization.

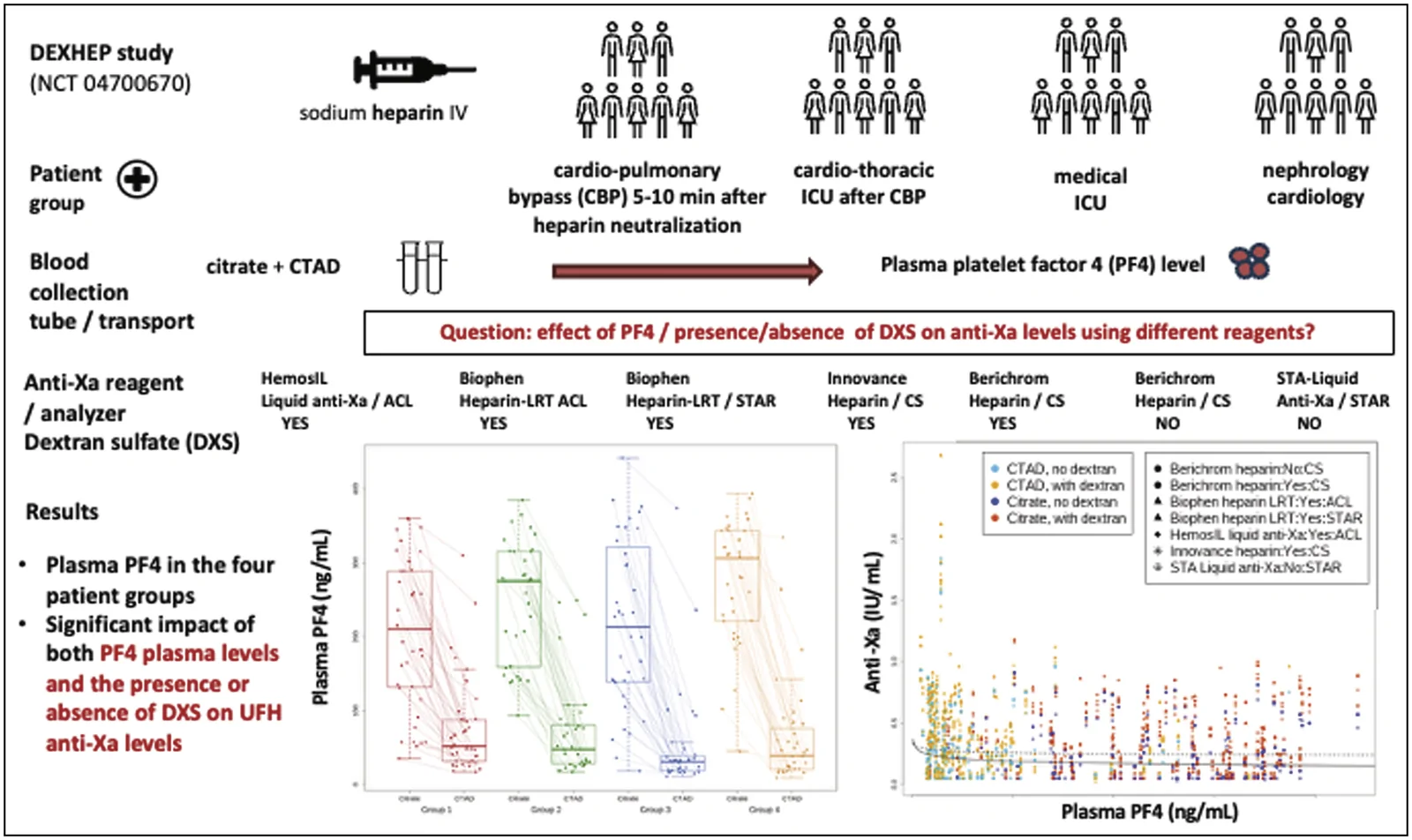

## Introduction

Management of patients on unfractionated heparin (UFH) therapy at therapeutic dose requires laboratory monitoring for dose adjustments due to the narrow therapeutic index of UFH and substantial inter-individual variability. 1 Monitoring may rely on laboratory tests performed on platelet poor plasma, such as activated partial thromboplastin time (aPTT) or anti-Xa assay. 1 2 3 4 APTT is sensitive to many conditions unrelated to UFH. 2 3 4 5 Therefore, chromogenic anti-Xa assays should be preferred given their better specificity for anti-Xa drugs. However, substantial inter-assay variability has been previously reported. 4 5 6 To better understand this variability, our group and others have demonstrated that the use of dextran sulfate (DXS), which may or may not be present in the reagents depending on the anti-Xa kits used, can have an impact on the anti-Xa result, with DXS increasing anti-Xa levels. 7 8 9 10 11 12 13 14 15 16 DXS is a polysaccharidic macromolecule which has the ability to displace UFH from proteins other than antithrombin that bind UFH. 6 Among heparin binding proteins, platelet factor 4 (PF4, also called CXCL4) is a tetrameric chemokine released into plasma from platelet α-granules following platelet activation. 17 PF4 contains numerous positively charged amino-acid residues that participate in the formation of complexes with negatively charged sulfate and carboxylate groups of UFH chains; these electrostatic interactions lead to the partial neutralization of UFH in vivo in different clinical situations, but also ex vivo, i.e., in blood collection tubes, and finally in vitro. 17 18 19 20 21 To the best of our knowledge, the relationship between plasma PF4 and anti-Xa levels measured using different reagent kits has never been specifically investigated. The aim of the present study was to investigate whether PF4 plasma levels along with the presence or absence of DXS in anti-Xa reagents are associated with variations in UFH anti-Xa levels in different settings. We then studied the agreement between two anti-Xa assays (Liquid-anti-Xa/DXS-free vs. Biophen-LRT/DXS) and their potential relevance in clinical decision-making regarding UFH dose adjustment at therapeutic levels.

## Methods

DEXHEP study (NCT04700670) was a prospective non-interventional study including patients receiving UFH (heparin sodium, Panpharma or Choay) from intensive care units or medical wards of eight French hospital centers between January 2020 and November 2021. 14 Briefly, four groups of patients were predefined: group 1 (post-cardiopulmonary bypass [CPB], 5–10 minutes after heparin neutralization by protamine), group 2 (cardiothoracic intensive care unit, Day 1 to Day 5 post-CPB), group 3 (medical intensive care unit), and group 4 (medical inpatients including patients undergoing coronary angioplasty or renal dialysis). The study protocol was approved by an Ethics Committee (Comité Consultatif de Protection des Personnes 19.03.28.40218). All enrolled patients or their relatives gave their informed consent to participate in the study.

Blood was collected in parallel in 0.109 M sodium citrate (Becton-Dickinson Vacutainer) and in citrate-theophylline-adenosine-dipyridamole (CTAD) tubes (Greiner-Bio-One Vacuette), designed to prevent platelet activation and granule release in collection tube. 21 22 Blood samples were transported to laboratories either by a pneumatic transport system (PTS) or by courier. Plasma samples were double-centrifuged as recommended and stored at −70°C in 500 µL aliquots. 23 Overall, centrifugation was performed within 1 or 2 hours of blood collection for 73 and 87% of plasma samples, respectively 14 ; freezing was performed within 10to 60 minutes following centrifugation. Plasma aliquots were shipped on dry ice for centralized measurement of UFH anti-Xa and PF4 levels. Anti-Xa levels were assessed using different reagent/coagulometer combinations with dedicated calibrators and controls as recommended by the manufacturers. 14 We used four reagents containing DXS (HemosIL-Liquid-anti-Xa-Werfen on ACL TOP-Werfen, Biophen Heparin-LRT-Hyphen-Biomed on STARMax-Stago and ACL-TOP-Werfen, Innovance-Heparin-Siemens on Sysmex-CS, and Berichrom-Heparin-Siemens on Sysmex-CS) and two without DXS (STA-Liquid-anti-Xa Stago on STARMax-Stago and Berichrom-Heparin-Siemens on Sysmex-CS) (seven reagent/automate combinations). PF4 was measured at Dijon University hospital in both citrate and CTAD samples (diluted 1:20) using Zymutest-PF4-kit (Hyphen-Biomed), following manufacturer's instructions (lower limit of quantification [LLOQ]: 12 ng/mL). Statistical analysis was performed on R-software (version 4.0.2 ref CORE TEAM) using the lme4 package (version 1.1.28). 24 25 Quantitative data were described as median (interquartile range), geometric mean values, and minimal and maximal values. The anti-Xa activity level was modeled using a linear mixed-effects model, after logarithmic transformation, using the presence of DXS and the PF4 level as covariates; an interaction term was included in the model, and the patient effect was included as a random effect on the intercept. Errors and random effects were assumed to be Gaussian and independent. Assumptions were checked graphically. Nested-models likelihood ratio tests were used to test for the effects of the predictors in the model, using the asymptotic chi-square distribution. All analyses were performed with a Type I error of 5%. The agreement between the results of anti-Xa assays in the presence or absence of DXS was evaluated graphically.

## Results

### PF4 Plasma Levels

Of the 165 patients included in DEXHEP study, 144 (mean age 66.7 years, 45 females and 99 males) were analyzed in the present study; available plasma volume was insufficient in 21 patients to measure PF4. The average PF4 levels in citrate plasma samples were consistently higher than in CTAD ones (206 ng/mL vs. 45.8 ng/mL, +351%, 95% CI [+301; +406]; *p* < 10 ^−4^ ) in the four patient groups ( Fig. 1 ). These results were observed when blood samples were transported by courier (citrate 183 ng/mL vs. CTAD 35 ng/mL, +424% [+349; +512], *p* < 10 ^−4^ ) or by PTS (citrate 241 ng/mL vs. CTAD 65 ng/mL, +269% [+212; +336], *p* < 10 ^−4^ ), with significantly higher levels using PTS. A significant positive correlation was observed between PF4 levels and the time elapsed from sampling to centrifugation for both citrate ( *r*_S_ = 0.35, *p* < 10 ^−4^ ) and CTAD ( *r*_S_ = 0.29, *p* = 4.10 ^−4^ ) plasma samples. A positive correlation was also found between PF4 levels and platelet count in citrate samples ( *r*_S_ = 0.47, *p* < 1.10 ^−4^ ), but not in CTAD samples ( *r*_S_ = 0.04, *p* = 0.6779).

**Fig. 1:**
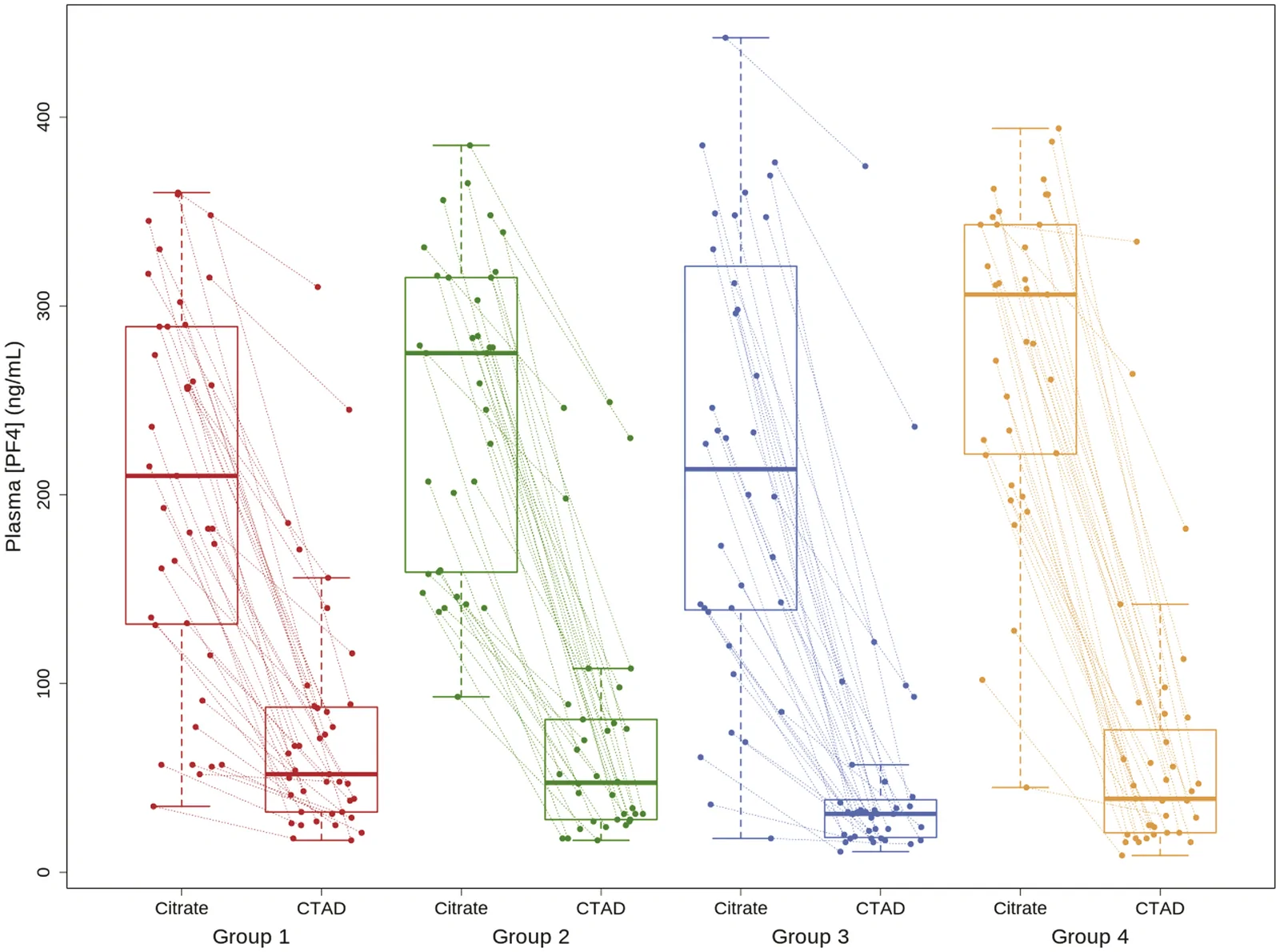
Plasma PF4 concentrations in citrate and CTAD samples represented in each group as box plots (median, 1st and 3rd quartiles, min-max). Group 1 (red): post-cardiopulmonary bypass (CPB), 5 to 10 minutes after heparin neutralization by protamine; group 2 (green): post-CPB surgery (Day 1 to Day 5); group 3 (blue): medical intensive care. ECMO; group 4 (yellow): medicine (cardiology, nephrology, internal medicine).

### Impact of DXS and PF4 on Anti-Xa Activity Levels

As we previously reported, 14 consistently higher levels of anti-Xa were observed with DXS-containing reagents than with DXS-free reagents, irrespective of the use of citrate or CTAD tubes to collect blood ( Table 1 ). Moreover, anti-Xa levels were consistently higher in CTAD samples than in citrate samples, irrespective of patient group and anti-Xa assay.

**Table 1:** Anti-FXa values measured with reagents containing or not DXS, in citrate or CTAD plasma samples a

Reagent	Patient groups									
	Group 1 CPB, 5–10 min after heparin neutralization by protamine		Group 2 Intensive care unit 1–5 days post CPB		Group 3 Medical intensive care unit		Group 4 Other medical patients		All groups	
	Citrate	CTAD	Citrate	CTAD	Citrate	CTAD	Citrate	CTAD	Citrate	CTAD
**Reagents with DXS**										
**Berichrom Heparin** **Siemens (IU/mL)**	*n* = 39 * m _e_* = 0.34 [0.05; 1.36]	*n* = 39 * m _e_* = 0.30 [0.05; 1.24]	*n* = 34 * m _e_* = 0.08 [0.05 ; 0.67]	*n* = 34 * m _e_* = 0.1 [0.05 ; 0.67]	*n* = 36 * m _e_* = 0.33 [0.05 ; 2.16]	*n* = 36 * m _e_* = 0.34 [0.08 ; 2.02]	*n* = 35 * m _e_* = 0.61 [0.05 ; 1.58]	*n* = 35 * m _e_* = 0.6 [0.05 ; 1.66]	*n* = 105 * m _e_* = 0.25 [0.05 ; 2.16]	*n* = 105 * m _e_* = 0.265 [0.05 ; 2.02]
**Biophen heparin LRT Hyphen (IU/mL)**	*n* = 78 * m _e_* = 0.3 [0.05; 1.6]	*n* = 78 * m _e_* = 0.3 [0.09; 1.52]	*n* = 68 * m _e_* = 0.11 [0.05 ; 0.71]	*n* = 68 * m _e_* = 0.155 [0.05 ; 0.61]	*n* = 72 * m _e_* = 0.38 [0.05 ; 2.6]	*n* = 72 * m _e_* = 0.43 [0.05 ; 2.68]	*n* = 70 * m _e_* = 0.575 [0.05 ; 2.16]	*n* = 70 * m _e_* = 0.62 [0.05 ; 2.12]	*n* = 210 * m _e_* = 0.3 [0.05 ; 2.6]	*n* = 210 * m _e_* = 0.36 [0.05 ; 2.68]
**HemosIL liquid anti-Xa - Werfen (IU/mL)**	*n* = 39 * m _e_* = 0.4 [0.05; 1.28]	*n* = 39 * m _e_* = 0.45 [0.05; 1.25]	*n* = 34 * m _e_* = 0.075 [0.05 ; 0.51]	*n* = 34 * m _e_* = 0.16 [0.05 ; 0.54]	*n* = 36 * m _e_* = 0.38 [0.05 ; 1.76]	*n* = 36 * m _e_* = 0.4 [0.05 ; 1.71]	*n* = 35 * m _e_* = 0.59 [0.05 ; 1.58]	*n* = 35 * m _e_* = 0.6 [0.02 ; 1.59]	*n* = 105 * m _e_* = 0.285 [0.05 ; 1.76]	*n* = 105 * m _e_* = 0.345 [0.02 ; 1.71]
**Innovance heparin** **Siemens (IU/mL)**	*n* = 39 * m _e_* = 0.27 [0.05; 1.22]	*n* = 39 * m _e_* = 0.31 [0.05; 1.29]	*n* = 34 * m _e_* = 0.095 [0.06 ; 0.46]	*n* = 34 * m _e_* = 0.13 [0.09 ; 0.49]	*n* = 36 * m _e_* = 0.32 [0.05 ; 1.5]	*n* = 36 * m _e_* = 0.34 [0.05 ; 1.34]	*n* = 35 * m _e_* = 0.48 [0.05 ; 1.49]	*n* = 35 * m _e_* = 0.47 [0.05 ; 1.45]	*n* = 105 * m _e_* = 0.23 [0.05 ; 1.5]	*n* = 105 * m _e_* = 0.29 [0.05 ; 1.45]
***All with DXS*** **(IU/mL)**	*n* = 195 * m _e_* = 0.32 [0.05; 1.60]	*n* = 195 * m _e_* = 0.34 [0.05; 1.52]	*n* = 170 * m _e_* = 0.1 [0.05 ; 0.71]	*n* = 170 * m _e_* = 0.14 [0.05 ; 0.67]	*n* = 180 * m _e_* = 0.37 [0.05 ; 2.6]	*n* = 180 * m _e_* = 0.38 [0.05 ; 2.68]	*n* = 175 * m _e_* = 0.55 [0.05 ; 2.16]	*n* = 175 * m _e_* = 0.585 [0.02 ; 2.12]	*n* = 525 * m _e_* = 0.28 [0.05 ; 2.6]	*n* = 525 * m _e_* = 0.33 [0.02 ; 2.68]
**Reagents without DXS**										
**Berichrom heparin** **Siemens (IU/mL)**	*n* = 39 * m _e_* = 0.05 [0.05; 0.57]	*n* = 39 * m _e_* = 0.05 [0.05; 0.52]	*n* = 34 * m _e_* = 0.095 [0.05 ; 0.35]	*n* = 34 * m _e_* = 0.14 [0.05 ; 0.42]	*n* = 36 * m _e_* = 0.3 [0.05 ; 1.84]	*n* = 36 * m _e_* = 0.32 [0.05 ; 1.8]	*n* = 35 * m _e_* = 0.48 [0.05 ; 1.56]	*n* = 35 * m _e_* = 0.59 [0.06 ; 1.56]	*n* = 105 * m _e_* = 0.22 [0.05 ; 1.84]	*n* = 105 * m _e_* = 0.275 [0.05 ; 1.8]
**STA Liquid anti-Xa** **Stago (IU/mL)**	*n* = 39 * m _e_* = 0.05 [0.05; 1.62]	*n* = 39 * m _e_* = 0.05 [0.05; 1.46]	*n* = 34 * m _e_* = 0.05 [0.05; 0.3]	*n* = 34 * m _e_* = 0.05 [0.05; 0.35]	*n* = 36 * m _e_* = 0.22 [0.03 ; 1.75]	*n* = 36 * m _e_* = 0.265 [0.05 ; 1.59]	*n* = 35 * m _e_* = 0.36 [0.04 ; 1.75]	*n* = 35 * m _e_* = 0.49 [0.05 ; 1.84]	*n* = 105 * m _e_* = 0.14 [0.03 ; 1.75]	*n* = 105 * m _e_* = 0.2 [0.05; 1.84]
***All without DXS*** **(IU/mL)**	*n* = 78 * m _e_* = 0.05 [0.05; 1.62]	*n* = 78 * m _e_* = 0.05 [0.05; 1.46]	*n* = 68 * m _e_* = 0.06 [0.05; 0.35]	*n* = 68 * m _e_* = 0.12 [0.05; 0.42]	*n* = 72 * m _e_* = 0.27 [0.03; 1.84]	*n* = 72 * m _e_* = 0.28 [0.05; 1.8]	*n* = 70 * m _e_* = 0.44 [0.04; 1.75]	*n* = 70 * m _e_* = 0.54 [0.05 ; 1.84]	*n* = 210 * m _e_* = 0.16 [0.03; 1.84]	*n* = 210 * m _e_* = 0.24 [0.05; 1.84]

Abbreviations: CPB, cardiopulmonary bypass; *n* , number of values, *m _e_* , median; [min; max].

Notes: By convention all values <0.1 IU/mL were taken as 0.05 IU/mL.

a Only results from patients in whom platelet factor 4 could be measured are presented here.

Since patients in group 1 had a high proportion (up to 77%) of anti-Xa results under the LLOQ (<0.1 IU/mL) due to heparin neutralization by protamine ( Table 1 ), and given the specific issue of protamine–heparin dissociation, we further focused on patients in the other three groups: 2, 3, and 4.

Using a linear mixed-effect model, we found a significant effect of both DXS and PF4 on anti-Xa levels, with a significant interaction term ( *p* < 10 ^−4^ ), meaning that the effect of DXS on anti-Xa level depended on the PF4 level. Fig. 2 shows the relationship between PF4 and anti-Xa levels using reagents containing or not DXS: increasing PF4 levels decrease the average anti-Xa levels, to a slightly greater extent when using DXS-free reagents (−0.14 IU/mL, 95% CI [−0.18; −0.11], *p* < 10 ^−4^ ) than when using DXS-containing assays (−0.06 IU/mL, 95% CI [−0.08; −0.03], *p* < 10 ^−4^ ). The median bias related to the presence of DXS was 0.05 IU/mL at low PF4 levels (<30 ng/mL), rising to 0.09 IU/mL at high PF4 levels (beyond 300 ng/mL).

**Fig. 2:**
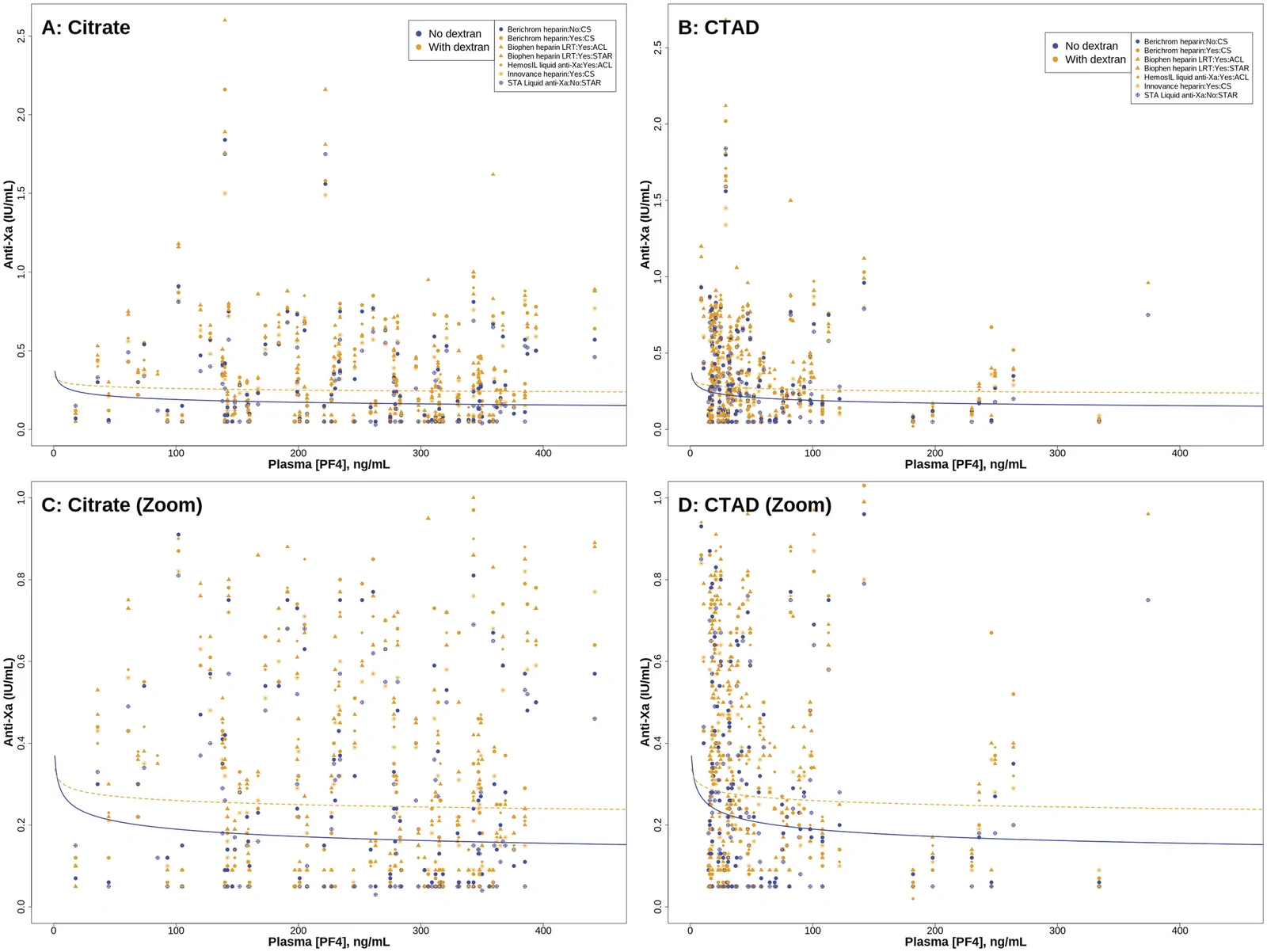
Anti-Xa activity results measured with the different reagent/coagulometer combinations as a function of PF4 concentration in groups 2 to 4. Results obtained in citrate tubes ( **A** , **C** ) and CTAD tubes ( **B** , **D** ). (C D) A zoom on anti-Xa levels comprised between <0.10 and 1.0 IU/mL. Each symbol represents a reagent/coagulometer combination (for details, see the insert). Vertically aligned symbols correspond to a patient and a tube (citrate or CTAD) associated with a given PF4 concentration. Blue symbols correspond to reagents not containing DXS; orange symbols correspond to DXS reagents. The continuous blue curves represent for a median patient the median value of anti-Xa using reagents without DXS, the dashed orange curves the median value using reagents with DXS.

We also wondered whether the use of CTAD tubes rather than citrate tubes could limit this phenomenon since CTAD is aimed at limiting platelet activation. Up to differences in plasma PF4 levels between the two collection tubes of around 200 ng/mL, the bias between anti-Xa levels was rather small (<0.05 anti-Xa IU/mL) and constant whether or not the reagent contained DXS, anti-Xa being consistently lower in citrate tubes compared with CTAD tubes ( Fig. 3A ). When PF4 level differences exceeded roughly 200 ng/mL, biases in anti-Xa levels increased progressively with all reagents except one, the Berichrom-Heparin containing DXS; for PF4 level differences exceeding 250 to 300 ng/mL, i.e., in a minority of patients, the bias reached aound 0.1 IU/mL. Noteworthy, Berichrom-Heparin was the only assay we were able to test in the presence or absence of DXS. In the presence of DXS, this reagent showed negligible constant bias between citrate and CTAD tubes, even at high PF4 differences ( Fig. 3B ); in the absence of DXS, an increasing bias was observed ( Fig. 3C ). These results suggest that DXS effectively dissociated heparin from PF4 whatever the PF4 concentration. In contrast, the higher anti-Xa levels in CTAD than in citrate samples with all other DXS-containing reagents suggest that DXS failed to fully dissociate complexes at high PF4 levels ( Fig. 2 ). These discrepant results suggest that type and/or concentration of DXS used in reagents may impact their ability to fully dissociate complexes.

**Fig. 3:**
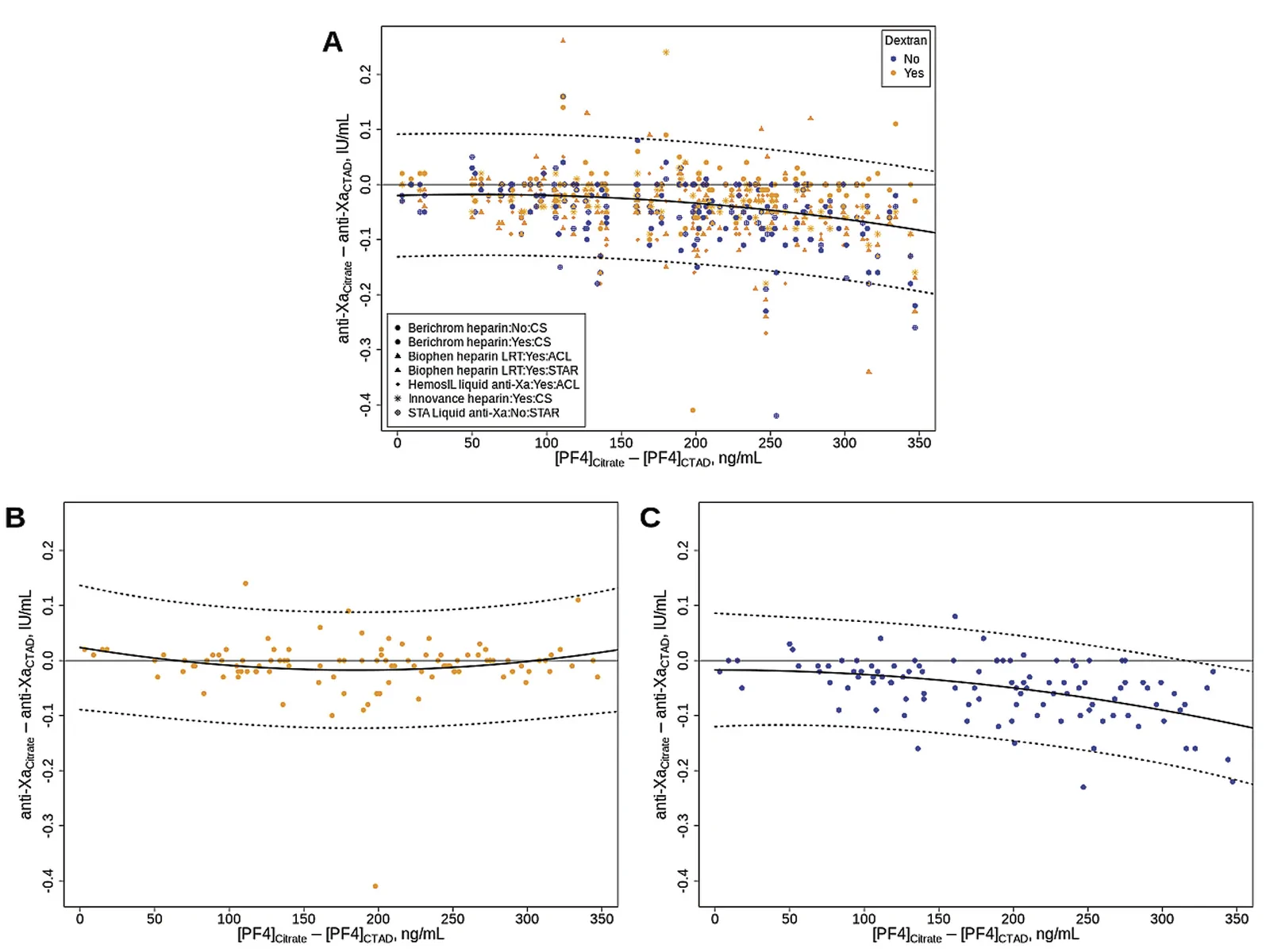
Bias (anti-Xa value measured in citrate tube minus measured in CTAD tube) as the difference of PF4 plasma concentration in groups 2 to 4. Values obtained using reagents without DXS are represented in blue, with DXS in orange. The continuous black curve represents the median bias, while grey lines represent the 95% prediction interval. ( **A** ) All methods: each symbol represents a reagent/coagulometer combination (for details, see the insert); ( **B** ) Berichrom with DXS. ( **C** ) Berichrom without DXS.

### Agreement Between Anti-Xa Levels (STA-Liquid-anti-Xa vs. Biophen-LRT)

To further investigate the relevance of anti-Xa differences in patient groups 2, 3, and 4, we studied the agreement between anti-Xa levels measured using STA-Liquid-anti-Xa (without DXS) and Biophen LRT (containing DXS), in citrate and CTAD tubes, all measured using a STARMax analyzer ( Fig. 4 ; Table 2 ). We chose those two assays because they showed the greatest differences in anti-Xa levels. Considering the 0.3 to 0.7 IU/mL therapeutic range, agreement between anti-Xa values was obtained in 66/102 patients (65%) using citrate tubes and 69/101 patients (68%) using CTAD tubes. In other words, disagreement between assays in the presence or absence DXS was observed in 35 and 32% using citrate and CTAD tubes, respectively. Consequently, a substantial proportion of patients had anti-Xa results below the therapeutic interval using the DXS-free reagent, but within the therapeutic range or above using the DXS reagent. Conversely, patients had results above the therapeutic interval using the DXS reagent ( Fig. 4 ).

**Fig. 4:**
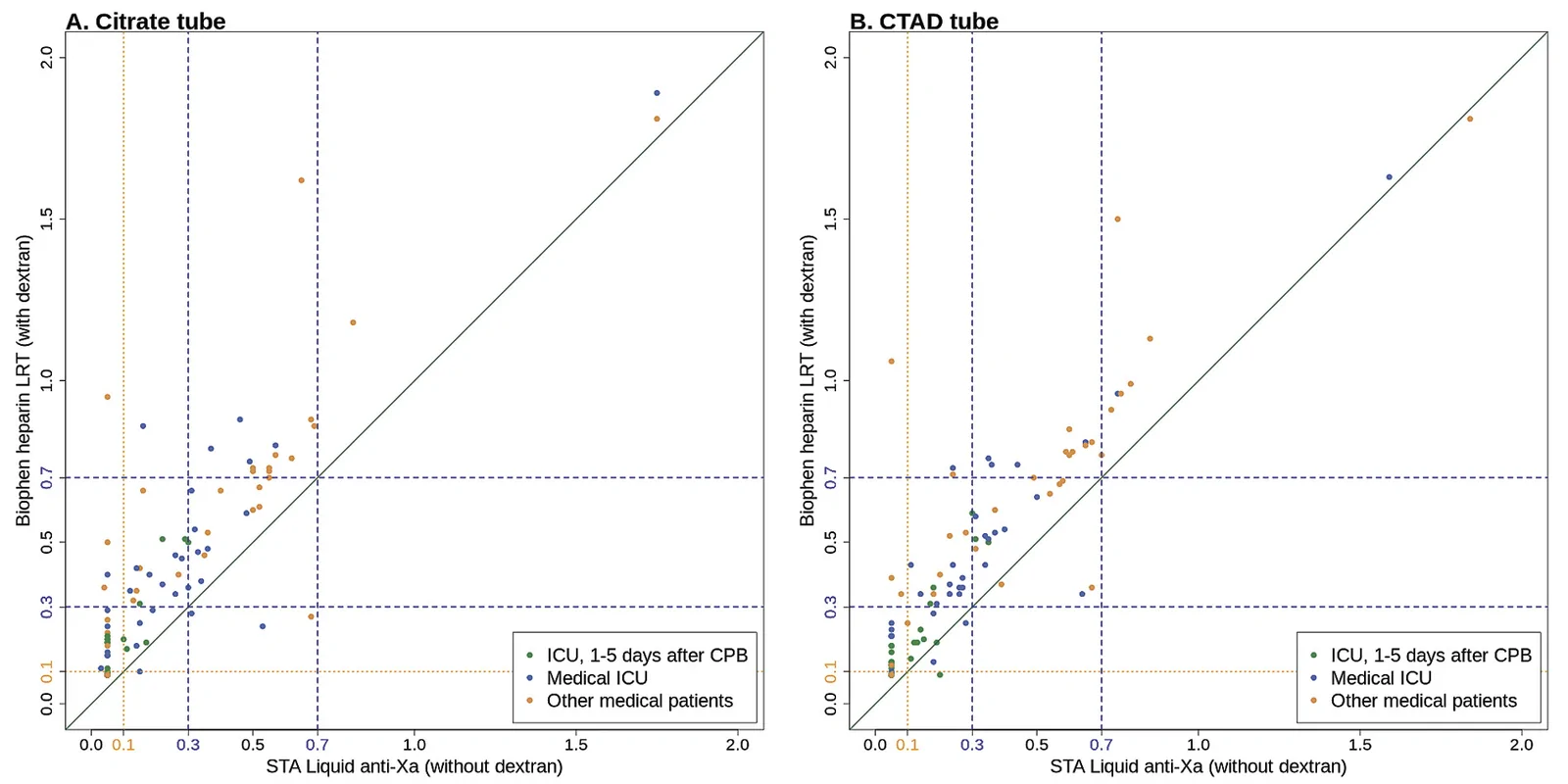
Agreement between anti-Xa values measured on STARMax system (Stago) with STA Liquid-anti-Xa Stago (without DXS) and Biophen LRT (Hyphen) (with DXS) in groups 2 to 4. Blue dashed lines illustrate different anti-Xa thresholds used in clinical practice for unfractionated heparin (UFH) dose adjustment. Yellow dashed lines illustrate the limit of quantification. ( **A** ) Citrate tube. ( **B** ) CTAD tube.

**Table 2:** Agreement between anti-Xa levels measured using STA-Liquid-anti-Xa (DXS free) and Biophen LRT (containing DXS) in citrate and CTAD tubes

		Anti-Xa (IU/mL) with DXS Biophen heparin LRT				Anti-Xa (IU/mL) with DXS Biophen heparin LRT			
		Citrate tubes				CTAD tubes			
		<0.3	[0.3–0.7]	> 0.7	Total	<0.3	[0.3–0.7]	>0.7	Total
**Anti-Xa (IU/mL)** **DXS-free** **STA Liquid anti-Xa**	**<0.3**	**48**	**18**	**2**	**68**	**42**	**18**	**3**	**63**
	**[0.3** – **0.7]**	**3**	**15**	**13**	**31**	**0**	**19**	**11**	**30**
	**>0.7**	**0**	**0**	**3**	**3**	**0**	**0**	**8**	**8**
**Total**		**51**	**33**	**18**	**102**	**42**	**37**	**22**	**101**

## Discussion

This is the first study to demonstrate a significant impact of both PF4 plasma levels and the presence or absence of DXS in different anti-Xa reagents on UFH anti-Xa levels in different patient settings, showing consistently higher levels in the presence of DXS, along with a significant interaction between the effects of DXS and PF4 levels.

PF4 levels measured in plasma depend on in vivo and ex vivo platelet activation. In the present study, we first showed that PF4 levels measured in citrate tubes were correlated with platelet counts, as expected since PF4 is released from platelet α-granules. Second, compared with healthy subjects (<30 ng/mL in CTAD plasmas), relatively high levels of PF4 were found in the four patient groups we studied regardless of the collection tube: these results were consistent with the conditions of DEXHEP patients on UFH therapy for whom circuits with artificial surfaces were used (CPB, ECMO, angioplasty, renal dialysis, use of catheter for sampling…). 18 19 20 In addition, ex vivo platelet activation depends on multiple factors, including blood sampling depending on the difficulty of phlebotomy, tube transport (PTS activates platelets as we have observed), and the time elapsed between blood collection and centrifugation. 22 23 26 The roughly 3-fold higher PF4 levels in citrate tubes compared with CTAD, irrespective of the patient group we observed, suggest that ex vivo platelet activation is responsible for a large proportion of the PF4 plasma level when citrate tubes are used. We therefore confirm that CTAD tubes, containing the antiplatelet agent dipyridamole, effectively prevented PF4 release from platelets. 21 27 28 29 Furthermore, since most samples in our study were centrifuged within a short time after blood collection (1 hour in 73% of cases), the important differences in PF4 levels between citrate and CTAD tubes, irrespectively of the group, show that platelet activation occurred very early after blood collection process. Remarkably, PF4 levels in CTAD tubes remained low in the vast majority of patients whatever the group. Moreover in CTAD tubes, platelet count no longer influenced PF4 levels.

The increasing effect of DXS on UFH anti-Xa levels that we have observed in all groups of patients suggests that DXS dissociates complexes formed between heparin chains rich in polyanions and proteins rich in cationic residues via electrostatic interactions. 14 15 Indeed, PF4 is a highly cationic, tetrameric, globular protein of 32 kDa comprising two homodimers; the 70-amino acid monomer with 13 positive charges (2 histidine, 3 arginine, and 8 lysine residues—i.e., the 3 cationic amino acids) is key to the binding of PF4 of anionic heparin, forming a heparin-binding site located at what Warkentin et al called the “equator” around the “globe” (globular protein), forming a ring of positive charges. 17 30

Our approach, using CTAD and citrate collection tubes, allowed us to evaluate the relative impact of PF4 levels due to ex vivo platelet activation on anti-Xa results, depending on reagents, especially their DXS content using the Berichrom-Heparin reagent. The differences in PF4 levels between the two sample tubes could reach up to 300 to 350 ng/mL, indicating significant platelet activation in citrate tube: in such cases, DXS-free reagent lowered anti-Xa levels to approximately 0.1 IU/mL in citrate tubes, illustrating the effect of ex vivo activation due to the sampling process. Noteworthy, such PF4 differences were observed in a minority of our patients. In contrast, when the difference in PF4 levels between citrate and CTAD tubes was less than 200 ng/mL, i.e., as in most cases, the difference in anti-Xa levels was negligible (<0.05 IU/mL), and therefore of limited clinical relevance. Overall, our results suggest that provided the blood sample is carefully drawn and processed, thus minimizing platelet activation and subsequently PF4 release, the underestimation of anti-Xa levels is weak, consistent with the results of previous studies comparing citrate and CTAD collection tubes. 27 28 29

Interestingly, the Berichrom-Heparin reagent we were able to use with or without DXS showed that, in this particular case, DXS allowed anti-Xa levels to be obtained independently of PF4 levels. In contrast, with all other DXS-containing assays, DXS failed to fully recover heparin from complexes with PF4 when PF4 levels were high. Such differences suggest that the effect of DXS likely depends on the type and/or the concentration of the polysaccharidic macromolecule added in the reagents. Interestingly, using in vitro spiking experiments, Hardy demonstrated that increasing concentrations of DXS (8,000 Da; from 6,500 to 10,000—sulfur content: 16 to 20%) increased anti-Xa levels measured with Biophen heparin LRT reagent. 15 Unfortunately, DXS type and/or concentration are rarely specified by manufacturers.

Finally, we demonstrated that differences in anti-Xa levels related to the presence or absence of DXS in two assays could lead to different treatment decision-making, which is in agreement with recent previous studies conducted in other settings such as ECMO. 6 13 16 When considering a given therapeutic interval, for instance 0.3 to 0.7 IU/mL, patients had anti-Xa levels below the therapeutic interval using the DXS-free reagent, but within or above it using the DXS reagent. Conversely, patients had results above the therapeutic interval using the DXS reagent, but within the therapeutic interval using the DXS-free reagent: our results are consistent with those of the literature. 6 13 16 It must be remembered that the therapeutic range of anti-Xa level 0.3 to 0.7 IU/mL was determined with DXS-free reagent. 31 This raises the question of redefining therapeutic intervals in the different patient settings using current anti-Xa reagents.

Our study has some limitations. First, we were able to test only one reagent with and without DXS, and only two reagents without DXS. Second, we could not evaluate the impact of the observed disagreement in the management of patients regarding dose adjustments or in terms of clinical outcomes (bleedings, thrombosis). Further studies are needed to investigate this issue.

In conclusion, PF4 levels may affect anti-Xa levels but do not fully explain the differences of anti-Xa levels observed in the presence or absence of DXS. Anti-Xa assays require standardization. Not all anti-Xa assays containing DXS are equivalent, and more information is needed to better understand the differences observed.

## Notes

**What is known about this topic?**

- Inter-assay variability in anti-Xa levels for heparin monitoring can be explained in part by the presence or absence of dextran sulfate (DXS) in the reagents.
- DXS has the ability to dissociate UFH from neutralizing proteins, including platelet factor 4 (PF4).

**What does this paper add?**

- We evaluated plasma PF4 and UFH anti-Xa level using seven reagent/analyzer combinations (two without dextran) on citrate and CTAD samples from 144 patients from different settings.
- PF4 levels slightly affect anti-Xa levels, the use of CTAD tubes minimizing the effect.
- PF4 levels do not fully explain the differences of anti-Xa levels observed in the presence or absence of DXS, which has a greater effect.
